# Aspects of the Epigenetic Regulation of EMT Related to Cancer Metastasis

**DOI:** 10.3390/cells10123435

**Published:** 2021-12-06

**Authors:** Ewa Nowak, Ilona Bednarek

**Affiliations:** Department of Biotechnology and Genetic Engineering, Faculty of Pharmaceutical Sciences in Sosnowiec, Medical University of Silesia, 40-055 Katowice, Poland; ibednarek@sum.edu.pl

**Keywords:** epithelial to mesenchymal transition (EMT), cancer metastasis, epigenetic regulation of EMT biomarkers

## Abstract

Epithelial to mesenchymal transition (EMT) occurs during the pathological process associated with tumor progression and is considered to influence and promote the metastatic cascade. Characterized by loss of cell adhesion and apex base polarity, EMT enhances cell motility and metastasis. The key markers of the epithelial to mesenchymal transition are proteins characteristic of the epithelial phenotype, e.g., E-cadherin, cytokeratins, occludin, or desmoplakin, the concentration and activity of which are reduced during this process. On the other hand, as a result of acquiring the characteristics of mesenchymal cells, an increased amount of N-cadherin, vimentin, fibronectin, or vitronectin is observed. Importantly, epithelial cells undergo partial EMT where some of the cells show both epithelial and mesenchymal characteristics. The significant influence of epigenetic regulatory mechanisms is observed in the gene expression involved in EMT. Among the epigenetic modifications accompanying incorrect genetic reprogramming in cancer are changes in the level of DNA methylation within the CpG islands and posttranslational covalent changes of histone proteins. All observed modifications, which are stable but reversible changes, affect the level of gene expression leading to the development and progression of the disease, and consequently affect the uncontrolled growth of the population of cancer cells.

## 1. Epithelial to Mesenchymal Transition

Epithelial to mesenchymal transition (EMT) is primarily involved in embryogenesis and is associated with adult tissue regeneration, wound healing, and fibrosis. During biological processes, EMT is a highly regulated process, while the other type of EMT occurring during tumor progression is deregulated and transient [1]. During transition of epithelial cells, loss of apical and basolateral polarity, disruption of cell adhesion, remodeling of the cytoskeleton, and changes in cell to matrix adhesion are observed, and cells become migratory mesenchymal cells [2]. All of these processes are associated with changes in the expression of genes encoding proteins characterizing a specific cellular phenotype. These changes relate, for example, to genes encoding extracellular matrix components; cytoskeletal genes mediating cell adhesion, migration, motility, and morphogenesis; genes controlling cell differentiation, development, growth, and proliferation; as well as signal transduction and transcription factor genes that cause EMT and all of its associated processes [3,4]. The main characteristics of epithelial cells are gradually lost, and partial EMT occurs where some cells show both epithelial and mesenchymal characteristics. Those partial EMT states are more relevant to cancer metastasis than the defined mesenchymal phenotype, and the number of potential intermediate EMT states within a tumor can be an indicator of malignancy [5]. There is evidence that not all cells in the primary tumor change morphology towards the mesenchymal state and express EMT-TFs. It is important that malignization, understood as ability of tumor cells to disseminate, is not universal for all cells in a tumor. Some studies suggest that EMT is required for metastasis, while other studies suggest that EMT may be dispensable or irrelevant for metastasis. However, the effect of EMT on the tumorigenesis of different cancers including prostate, lung, liver, pancreatic, and breast cancers has been demonstrated [5,6]. 

EMT is reversible by the mesenchymal to epithelial transition (MET), which is thought to affect circulating cancer cells when they reach a desirable metastatic niche and settle down to proliferate and develop secondary tumors [7].

## 2. Cancer Metastasis

Cancer metastasis requires multiple steps, including local invasion, intravasation, and colonization of distant organs. Cytoskeletal rearrangements within the cancer cells, combined with the action of adhesive interactions, secreted extracellular matrix metalloproteinases (MMPs), and cathepsins, drive cancer cell invasion and migration through the stroma. Cancer cells may migrate as single cells boring a path through the extracellular matrix, move along collagen fibers, or migrate collectively as ensembles that forge ahead from the tumor invasion front [8].

Cancer cells disseminate from tumors by invading blood vessels and the lymphatic system. Hematogenous dissemination is the main form of metastatic spread to organs. Circulating tumor cells (CTCs) in the blood circulation express progenitor and epithelial to mesenchymal transition markers, suggesting that these cells are the beginning of metastatic tumors [4,9].

Genomic instability is the cause of tumor initiation and progression and occurs in a metastatically competent subclone of a primary tumor. Genomic changes are reflected in many cellular phenotypes, any one of which may have all the necessary properties to complete the metastatic process. The most predominant genes that are changed in metastasis include tumor protein p53 (*TP53*), cyclin-dependent kinase inhibitor 2A (*CDKN2A*), phosphatase and tensin homolog (*PTEN*), phosphatidylinositol-4,5-bisphosphate 3-kinase catalytic subunit alpha (*PIK3CA*), and retinoblastoma (*RB1*) [4].

## 3. Epigenetic Regulation of EMT Biomarkers

Molecular mechanisms governing EMT leading to a downregulation of epithelial markers and an increase in mesenchymal markers and EMT-linked transcription factors can depend on epigenetic modifications, and in certain cases are tissue-specific. Detailed literature data are provided in the Table 1. DNA methylation is one of the best characterized mechanisms of epigenetic gene regulation and may be involved in regulating EMT [10].

### 3.1. Epigenetic Changes of Genes Mediating Cell Adhesion, Migration, and Motility

The E-cadherin (*CDH1* gene encodes a classical cadherin of the cadherin superfamily, epithelial calcium-dependent adhesion protein, a component of the adherent junctions between epithelial cells with tumor suppressor activity) gene promoter, which possesses several regulatory sequences including three E-boxes that mediate CDH1 transcriptional repression in mesenchymal cells, is transcriptionally and epigenetically regulated during EMT [11]. Besides the promoter, there are seven regions in the *CDH1* gene with regulatory functions, and six of them are located in intron 2, which has been previously identified as a cis-regulatory element with a pivotal role in controlling the gene’s expression [35]. In addition, the methylation of CpG sites located in the CDH1 enhancers correlates with the low gene expression [35].

*CDH1* transcription may directly or indirectly depend on expression control of miRNAs and long noncoding RNAs, due to the number of pathways regulating *CDH1* expression through the activity of Snail, Slug, ZEB1/2, EZH2, and Twist transcription factors [36]. miR-101 has been reported to act as a tumor suppressor by targeting CDH1 inhibitors, such as ZEB1/ZEB2 and EZH2 in different tumors. miR-200 family members are known as transcriptional repressors of E-cadherin by targeting ZEB1/ZEB2, meanwhile elevating expression of β-catenin and relocating mostly membrane-associated β-catenin to nucleus, thus activating the Wnt/β-catenin signaling pathway. Aberrant activation of the Wnt/β-catenin signaling pathway promotes cell proliferation, metastasis, and tumorigenesis [37].

N-cadherin (*CDH2* gene encodes a classical cadherin and member of the cadherin superfamily, neuronal calcium-dependent adhesion protein) is the transmembrane protein that mediates cell adhesion and promotes cell migration and invasion, which makes it an important factor in the epithelial to mesenchymal transition (EMT) [38]. A defined cell–cell junction staining of N-cadherin was visible after transfection with miR-338-3p, which is epigenetically silenced in gastric cancer. The Ras related protein (Rab-14) and Hedgehog acyltransferase (Hhat) are direct targets of miR-338-3p and enforced expression of miR-338-3p, and small interfering RNA induced Rab14-mediated accumulation of N-cadherin in the cell junctions [39].

Fibronectin (*FN1* gene encodes fibronectin, a glycoprotein present in a soluble dimeric form in plasma, and in a dimeric or multimeric form at the cell surface and in extracellular matrix) is widely expressed by several cell types and is involved in the migration, proliferation, and adhesion of cells, and in changes of the extracellular matrix through signaling with integrins in several types of cancers [40]. Upregulation of *FN1* may depend on miR-9-3p downregulation in cancer cells. The antitumor role of miR-9-3p including suppressing cell proliferation, migration, and invasion via downregulating *FN1* (among others) and inactivating the EMT process was confirmed in nasopharyngeal carcinoma [16].

Matrix Metallopeptidase 2 (*MMP2*) is a member of the matrix metalloproteinase gene family, the zinc dependent enzymes capable of cleaving components of the extracellular matrix and molecules involved in signal transduction. Different statuses of epigenetic changes within the MMP2 promoter were observed in various neoplasms. *MMP2* promoter regions are hypermethylated in non-migratory breast cancer cells and hypomethylated in the highly migratory glioma cells [17,41].

For Matrix Metallopeptidase 9 (*MMP9*) the enzyme encoded by this gene degrades type IV and V collagens and is involved in the breakdown of the extracellular matrix during arthritis or metastasis. Changes in the degree of methylation, both within *CpG* DNA islands and histone proteins, have been observed in cancer with different expression levels of MMP9. The extensive CpG islands present in the coding region are epigenetically repressed in glioma cells. In breast carcinoma cells, the elevated expression of *MMP9* correlated with the presence of H3K4me3 methylation [41]. DNA promoter methylation is important for the regulation of *MMP9* expression, and H3K4me3 is high in the promoter DNA regions, showing the importance of these binding sites in *MMP9* transcription in breast cancer cell lines [19]. Hypermethylation at the CpG-2 intragenic region is observed with higher *MMP9* expression levels, and the *MMP9* intragenic methylation hotspot may be responsible for the *MMP9* overexpression in melanoma cell lines [42]. Additionally, miR-211 acted as a tumor suppressor and inhibited EMT by targeting *MMP9* expression and may be a potential target of gastric cancer treatment [20]. Importantly, *MMP9* is one of the downstream target genes of SMYD3 (SET and MYND domain containing protein 3), the histone H3-K4 di-/tri- and H4-K5-methyltransferase, and overexpression of SMYD3 can induce *MMP9* expression in human tumor models, e.g., gastric cancer [43]. 

Desmoplakin (*DSP*) encodes a protein that anchors intermediate filaments to the desmosomal plaques and is involved in the organization of the desmosomal cadherin plakoglobin complexes. Loss of desmoplakin as a tumor suppressor was reportedly attributed to invasive tumorigenic potential. Decreased expression of *DSP* in a majority of lung cancer cell lines and primary lung tumors caused by methylation in the promoter region and in intron 1 indicated that epigenetic regulation is responsible for gene silencing [21].

Collagens (fibrillar forming collagen *COL1A1* encodes the proalpha 1 chains of type I collagen whose triple helix comprises two alpha 1 chains and one alpha 2 chain), found in most connective tissues, are involved in tumor invasion and progression. Collagen type I is composed of three polypeptide chains transcribed from *COL1A1* and *COL1A2* gene. Epigenetic alterations of collagen genes have been reported in various neoplasms, and each gene is methylated in several human cancer cells with coordinately decreased collagen expression. Although *COL1A1* mRNA is usually upregulated in tumor tissues, there is a small group of tumors that has downregulated mRNA expression, mainly due to promoter methylation. These downregulated cases were correlated with poor overall survival in patients with hepatocellular carcinoma [23,44].

### 3.2. Epigenetic Changes of Genes Controlling Cell Differentiation and Proliferation

Keratins (KRTs) are a family of intermediate filament proteins that are responsible for the structural integrity of epithelial cells and play significant roles in the occurrence, progression, and metastasis of multiple cancers [45]. *KRT8* and *KRT19* are two oncogenes important in development of human cancers, with high expression and hypomethylated promoters [29]. 

*KRT5, KRT14*, and *KRT17* overexpression is related to poor survival across different types of cancers. H3K27me3 promotes *KRT14* gene expression by altering the recruitment pattern of its transcription factor Sp1 [25]. Epigenetic silencing of *KRT13* genes might be useful for the development of diagnostic markers and novel therapeutic approaches [24]. KRT15 is associated with tumorigenesis and has different expression characteristics across different types of cancer [46]. Decreased expression of *KRT18* may be dependent on DNA hypermethylation and may serve as a biomarker for the diagnosis of metastasis [26]. The CpGs in the promoter of the *KRT19* gene and the methylation levels of *KRT19* are significantly inversely associated with gene expression, which suggests that DNA methylation is a potential epigenetic regulator in malignancy [28]. Decreased methylation correlates with increased *KRT23* transcript expression, which is inducible with the demethylating agent (5-Aza-2′-deoxycytidine), suggesting a potential epigenetic regulation for *KRT23* [30].

Occludin (the *OCLN* gene) encodes an integral membrane protein that is required for cytokine-induced regulation of the tight junctions (TJs) (paracellular permeability membrane). The hallmark of epithelial cells and their subsequent loss from cancer cells tends to be considered as a direct effect of epithelial to mesenchymal transition (EMT). *OCLN* can be partially methylated in the promoter sequence, and miR-122 has been reported to regulate gene expression [31].

Vimentin (the *VIM* gene encodes a type III intermediate filament protein, which is responsible for maintaining shape and integrity of the cytoplasm and stabilizing cytoskeletal interactions) plays a significant role in adhesion, migration, and signaling and is considered as a classic mesenchymal cell marker. *VIM* may be used for the early detection of several cancers and hypermethylation of the promoter is important for the transcriptional silencing of the gene in cancer cells [32]. On the other hand, increased levels of vimentin in the serum may be caused by hypomethylation of the vimentin gene and increased expression of the vimentin gene intracellularly, leading to secretion of high levels of soluble vimentin in the serum [34]. Additionally, varying degrees of methylation within a gene’s CpG islands leads to various levels of gene silencing, and DNA methylation is considered as an epigenetic biomarker for cancer detection [47]. *VIM* methylation seems to be a possible biomarker for the detection of tumor DNA in the serum of different colorectal carcinoma patients [33].

### 3.3. Transcription Factor Genes

EMT can be attained through cooperative functioning of different transcription factors such as SNAI1/2, TWIST, and ZEB1/2 with others (FOX, SOX, PRX, and HMGA2), leading to cell-forward consensus differentiations that make tumor cells capable of motility and metastasis. SNAIL1 (Snail) and SNAIL2 (Slug) activation downregulates the expression of genes involved in tight junctions (occludin and claudin 1), apical polarity (Crumbs Cell Polarity Complex Component 3, *CRB3*), and other cell adhesions such as E-cadherin, but upregulates the expression of N-cadherin, fibronectin, vimentin, and MMPs through different signaling pathways. Expression of E-cadherin, β-catenin, and γ-catenin is masked by TWIST1, but the expression of vimentin is increasing. Importantly, even a strong EMT-inducing transcription factor operates differentially in each cell type belonging to a different EMT status [48,49].

SNAI1 (Snail family transcriptional repressor 1) is the member of the SNAIL family that is the best studied EMT transcription factor and is predominantly expressed in cell lines with an epithelial phenotype. It is reported that SNAI1 expression at the onset of EMT and other EMT factors are induced at later time points to strengthen a mesenchymal state. Induction of SNAI1 leads to repression of SNAI2 and simultaneous induction of ZEB1/2 transcripts at an early stage, highlighting that execution of this functional circuit preceded other molecular and phenotypical changes that can lead to EMT. Epigenetic repression of SNAI1 on its target genes has been shown to be carried out through recruitment of HDAC1/2 and corepressor SIN3A on its SNAG domain [50]. Overexpression of SNAI1 is a pathologically relevant event in human acute myeloid leukemia (AML) that contributes to impaired differentiation and enhanced proliferation of immature myeloid cells, and ectopic expression of SNAI1 in hematopoietic cells predisposes to AML development, which is mediated by the interaction with the histone lysine-specific demethylase 1A KDM1A/LSD1 [51]. Suppression of SNAIL1 mediated gene reprogramming by SETDB1 occurs through increased H3K9 methylation at the *SNAI1* gene promoter that represses its H3K9 acetylation imposed by activated Smad3/4 complexes. SETDB1 regulates the activity of Smad3 at the *SNAI1* gene through a balance between histone methylation and acetylation [52].

ZEB1 (Zinc finger E-box binding homeobox 1) acts as a transcriptional repressor that inhibits interleukin-2 gene expression and represses the E-cadherin promoter, which induces an epithelial to mesenchymal transition (EMT) by recruiting SMARCA4/BRG1 [53]. ZEB1 recruits the histone deacetylase HDAC1 or the methyltransferase DNMT1 to the *CDH1* promoter to maintain the hypermethylation status and inhibit the transcription. The chromatin modifier SETD1B, a histone methyltransferase of Lys4 at histone H3, is a direct transcriptional target of ZEB1, which regulates the expression pattern of the SETD1B in colorectal cancer cells. ZEB1 binds the promoter region of the SETD1B gene and SETD1B-dependent active H3K4me3 histone code seems to open the ZEB1 promoter, forming a positive feedback loop [54]. The characteristic epigenetic regulation of plasticity in lung cancer cells is the interaction between the miR-200/ZEB axis in non-small-cell lung cancer. The miR-200 DNA promoters were hypermethylated in the human lung adenocarcinoma PC9 cell line in the TGF-β1-induced EMT process. Treatment with 5-aza-2′-deoxycytidine reversed hypermethylation, and ZEB1/2 expression was restored, leading to reversal of EMT [55]. Further epigenetic regulation of *ZEB1* depends on the protein arginine methyltransferases PRMT1, which induce the EMT process in breast cancer cells by mediating the asymmetric dimethylation of arginine 3 of histone H4 (H4R3me2as) at the *ZEB1* promoter and activating its transcription [56]. UTX (Ubiquitously Transcribed Tetratricopeptide Repeat on chromosome X) is identified as a histone demethylase that specifically targets di- and tri-methyl groups on lysine 27 of histone H3 (H3K27me2/3), which was shown to epigenetically induce the expression of ZEB1 in brain metastases during the metastasis of lung adenocarcinoma to the brain [57].

TWIST1 (Twist family bHLH transcription factor 1) is a basic helix−loop−helix (bHLH) transcription factor that plays an important role in embryonic development. 

TWIST has been long considered as a transcription repressor of the EMT, and increasing the expression of TWIST1 is directly associated with tumor invasion and metastasis and mediates the loss of E-cadherin, a key epithelial marker, by binding to the promoter and blocking its transcription [58]. TWIST is responsible for inducing the formation of actin-rich invadopodia, which recruit MMP7, MMP9, and MMP14 to the leading edge where they degrade ECM and basement membranes, facilitating tumor invasion and metastasis. *TWIST1* expression can be activated in response to the methyltransferase MMSET (nuclear receptor SET domain, NSD protein), leading to an increase in H3K36me2 and H3K27me3 methylation and suggesting a direct role of MMSET in the regulation of *TWIST1* expression, in turn leading to an increase in migration and invasion, and changes in cell morphology and gene expression consistent with an epithelial to mesenchymal transition (EMT) [59]. The lysine acetyltransferase TIP60/KAT5 mediates K73/76 diacetylation of the *TWIST* motif function as diacetylation in the histone H4 mimic GK-X-GK motif recruits BRD4 (a member of the BET, bromodomain and extra-terminal domain) protein and related transcriptional components to the super-enhancer of its targeted genes during progression of basal-like breast cancer [58]. *TWIST1* is upregulated and FOXO3a (the forkhead box class O 3a, which belongs to the FOXO subclass of forkhead transcription factors) is downregulated in breast cancer tissues, and overexpression of *TWIST1* can reverse the effect of FOXO3a on proliferation, invasion, migration, and EMT. Additionally, the overexpression of FOXO3a or knockdown of *TWIST1* suppresses the proliferation, invasion, migration, and EMT, and FOXO3a suppresses the growth and metastasis of breast cancer by targeting TWIST1 in vivo [60]. EZH2 (Enhancer of zeste homolog 2), a histone methyltransferase and a catalytic component of PRC2, catalyzes tri-methylation of histone H3 at Lys 27 (H3K27me3), which was shown to epigenetically suppress the expression of *TWIST* in brain metastases during the metastasis of lung adenocarcinoma to the brain. However, the role of EZH2 has not been fully established in lung adenocarcinoma. The major role of EZH2 in oncogenesis is to engage in the proliferation and development rather than the EMT process of the cancer. EZH2 is an interesting target for anticancer therapy because of upregulation in multiple cancers [57].

FOX. Deregulation of FOX factors is correlated with carcinogenesis and tumor progression. The FOXA factors clustered together with epithelial genes that are involved in the formation of adherens and tight junctions and the loss of FOXA1, 2, 3 impair the structure and function of enhancers at the tumor suppressor genes *CDH1*, *EPHB3*, and *CDX2*, which are integral to the differentiation and tissue integrity of the intestinal epithelium. Conversely, expression of FOXA factors in cells with inactive *CDX2* and *EPHB3* enhancers led to chromatin opening and de novo deposition of the H3K4me1 and H3K27ac marks. In cellular EMT models, ectopically expressed SNAIL1 directly represses FOXA1 and triggers downregulation of all FOXA family members, suggesting that loss of FOXA expression promotes EMT [61]. FOXF2 is specifically expressed in most basal-like breast cells, and the deficiency enhances metastatic ability and induces epithelial to mesenchymal transition (EMT) of cells by upregulating the transcription of *TWIST1*, which is a transcriptional target of FOXF2 [62].

### 3.4. Hypoxia-Induced EMT

Due to the metabolic conditions of the cancer, hypoxia is a common phenomenon in most solid tumors. A hypoxic microenvironment triggers varied molecular responses, but importantly, hypoxia and EMT share a number of common signaling pathways. This fact prompted researchers to even distinguish the term hypoxia-induced EMT. Among other stress-regulated factors, hypoxia-inducible factors (HIFs) are known to be the master regulators of gene expression under oxygen-deprived conditions. Transcriptional regulation by HIF proteins is mediated by an HIF complex consisting of oxygen-dependent HIFα and is expressed constitutively as HIFβ subunits, which bind to the hypoxia-responsive element (HRE) present in the promoter region of target genes [63]. HIF1 provokes EMT through upregulating transcription factors or repressors, activating the EMT-associated signaling pathways, modulating EMT-associated inflammatory cytokines, and regulating other pathways such as epigenetic regulators. It has been shown that HIF1 induces EMT through the transcriptional control of E-cadherin, SNAIL, ZEB1, and TWIST [64]. Hypoxia affects EMT through regulating signaling pathways, modulating expression and signaling of transcription factors, and regulating miRNA and lncRNA networks associated with EMT **[65].** miR-205 is the most down-modulated miRNA in prostate cancer cells upon cancer-associated fibroblast stimulation, due to direct transcriptional repression by HIF1, a known redox-sensitive transcription factor. Ectopic miR-205 overexpression in prostate cancer cells counteracts EMT, thus impairing enhancement of cell invasion, acquisition of stem cell traits, tumorigenicity, and metastatic dissemination. Additionally, miR-205 inhibits tumor-driven activation of surrounding fibroblasts by reducing pro inflammatory cytokine secretion **[66].** During hypoxia, most lncRNAs (HALs) are upregulated. HIF could directly promote the expression of these hypoxia-inducible lncRNAs through binding to the HREs (hypoxia response elements) located in their promoter [67]. Tumor hypoxia seems to play an important role to consider as a driver of EMT, tumor heterogeneity, and tumor immune escape.

### 3.5. Immunological Aspects of EMT

Inflammation contributes significantly to tumor cell metastasis and has been established as a key inducer of EMT during the progression of cancer. Inflammatory cytokines such as TGFβ, TNFα, IL-1, IL-6, and IL-8 activate transcription factors such as SMAD, NF-κB, STAT3, SNAIL, TWIST, and ZEB, which are considered to be potent inducers of EMT in different cancer types [68]. It has been confirmed that IL-6 is involved in the epithelial to mesenchymal transition in head and neck tumor cells via the activation of the STAT3/SNAIL signaling pathway, and STAT3 knock down significantly reverses IL-6-mediated EMT changes [69,70]. There are also results indicating that TNF*α* and IL-1b (but to a lower extent) induce EMT properties in breast tumor cells [71]. Additionally, TNF-α promotes phenotypic transition and increases the expression of EMT markers compared with TGF-β1 alone, which leads to the assumption that a pro-inflammatory tumor microenvironment enriched in TNF-α may accentuate TGF-β1-induced EMT in colon cancer cells [72]. TGF-β1 in the tumor microenvironment secreted from tumor-associated macrophages (M2 subtype (M2-TAMs)) activates the SMAD2/3 pathway and influences the increase in the expression levels of SOX4 and SOX2, which results in elevation of the migration ability of the cells in vitro, by influencing the expression of genes associated with the EMT process in glioma progression [73]. EMT induced by SNAIL accelerates cancer metastasis due to enhanced invasive ability but also induction of multiple immunosuppression and immune resistance mechanisms including immunosuppressive cytokines, regulatory T cells, impaired dendritic cells, and cytotoxic T lymphocyte resistance. Murine and human melanoma cells with typical EMT features after SNAIL transduction induced regulatory T cells and impaired dendritic cells partly through thrombospondin-1 (TSP1) production [74]. Activated human T cells are involved in the synthesis of IL-6, TNF*α*, and TGF*β*, which induce the expression of mesenchymal proteins such as fibronectin, vimentin, and ZEB1 in inflammatory breast cancer cells [75]. The inflammatory tumor microenvironment (TME) associated with EMT of ‘mesenchymal’ lung adenocarcinoma and lung squamous cell carcinoma show an increased expression of all cytokines and growth factor genes compared to ‘epithelial’ non-small cell lung cancer (NSCLCs). Expression of EMT-inducing cytokines IFN-γ and TNF-α are significantly increased in ‘mesenchymal’ cells, while expression of immunosuppressive cytokines IL-10 and TGF-β are also significantly increased in NSCLCs. TGF-β is not only immunosuppressive but also has EMT inducing and T-cell inhibiting functions [76]. It has an important role in the phenomenon of epithelial to mesenchymal transition (EMT), a phenotypic switch observed with carcinomas that promotes progression towards metastasis in relation to cancer inflammation [77].

## 4. Epidrugs in Cancer Therapy

DNA methylation is directly linked with histone deacetylation. DNA hypermethylation and hypoacetylation of histones H3 and H4 are linked to cancer progression. As a result, clinical studies have focused on inhibitors of DNA methyltransferases (DNMT) and HDAC inhibitors as a potential therapeutic approach to reverse cancer-promoting epigenetic changes [78]. There is evidence that some epidrugs play a significant role in the regulation of global DNA hypomethylation, which represses EMT [79]. There are data indicating that DNA methylation causally underlies EMT-driven resistance to cancer treatment, rather than being a mere consequence, e.g., several cell lines derived from various cancer types, including hepatocellular carcinoma, pancreatic, lung and ovarian cancer, acquired resistance to cancer therapies by activating an EMT. Importantly, after 5-aza-2′-deoxycytidine-mediated DNA demethylation, treatment-resistant cells lost their growth advantage under therapeutic pressure compared to control cells [80]. The first-generation of epidrugs included DNA methyltransferase inhibitors (the nucleoside analogs of DNMTi: 5-azacytidine, AZA [81], Vidaza^®^; 5-aza-2′-deoxycytidine, decytabine [82], Dacogen^®^), and histone deacetylase inhibitors (HDACi, the hydroxamic acids: suberoylanilide hydroxamic acid, SAHA, vorinostat [83], Zolinza^®^; trichostatin A, TSA [84]; cyclic peptides: trapoxin A, FK228, romidepsin [85], Istodax^®^) with a low degree of selectivity, and showed undesirable pharmacokinetic properties, poor target selectivity, and low bioavailability, were more active within non-physiological pH ranges, and are targets of cellular deaminases, which ultimately mean a short half-life for these compounds. The second-generation of epidrugs have improved physiological properties, including DNA methyltransferase inhibitors (the nucleoside analogs of DNMTi: zebularine; guadecitabine, S110 or SGI-110 [79], and non-nucleoside analogs: hydralazine, procainamide, RG108), and histone deacetylase inhibitors (HDACi: hydroxamic acid: belinostat, Beleodaq^®^; dacinostat; panobinostat [85], Farydak^®^; quisinostat [86]; benzamides: etinostat; mocetinostat [81]; chidamide, Tucidinostat, Epidaza^®^; carboxylic acids: valproic acid [87]). The mechanism of interaction of the third generation of epidrugs corresponds to the assumption that epigenetic factors could write, delete, or read epigenetic marks in the form of protein complexes, which is essential for the design of highly selective epidrugs. The third generation of epidrugs includes histone methyltransferase inhibitors (HMTi, e.g., EZH2i: GSK126 [81], GSK2816126A; tazemetostat, Tazverik^®^), histone demethylase inhibitors (HDMi), and bromodomain and extra-terminal domain inhibitors (BETi) [78,88]. Previous results have supported the assumption that co-treatment of epigenetic modifying drugs: 5-azacitidine (an inhibitor of DNA methyltransferases (DNMTs)), GSK126 (an inhibitor of Enhancer Of Zeste Homolog 2 (EZH2)), and/or mocetinostat (an inhibitor of class I histone deacetylases (HDACs)), with overexpression of transcription factor EMT suppressor Grainyhead-like 2 (GRHL2), could induce MET to different extents in ovarian cancer cell lines with an intermediate EMT or a full EMT state [81]. A series of epigenetic drugs for EMT are being tested in trials. Decitabine and 5-azacytidine have been approved by the FDA for acute myeloid leukemia and myelodysplasia treatment. HDAC inhibitors and HDM inhibitors are still under testing. The effectiveness of the use of this group of drugs is especially carefully observed, especially taking into account some study results showing contradictory evidence about the stimulatory effect on EMT in tumor and metastasis [89,90]. This problem must be taken into account in the conducted research. The development of the most effective epidrugs remains an open question. 

## 5. Conclusions

Epithelial to mesenchymal transition is a highly dynamic process involved in the metastatic cascade, as well as in specific hallmarks of cancer, such as cancer cell stemness, therapy resistance, and immune evasion. There is still some controversy about the involvement of EMT in metastasis. Due to the mutated state in cancer cells, chromatin regulators very often have pro-metastatic effects and activate EMT. For example, previous studies have confirmed that PRC2 methyltransferase Enhancer of Zeste Homolog 2 (EZH2) positively regulates breast cancer cell EMT through interaction with *SOX4*. The stimulatory effect results from transcriptional repression of epithelial genes [91]. Disturbances in maintaining the balance of functional antagonism of chromatin modulators, often resulting from mutations, may have a different effect on EMT in cancer [92]. Due to the nature of epigenetic modifications, the possibility of modulating these changes and understanding their functions in EMT and metastasis will provide new insight into our understanding of tumor progression and metastasis. Owing to the reversible nature of epigenetic modifications, a thorough understanding of their functions in EMT provides new insights into our knowledge of cancer progression and metastasis. Moreover, it can further facilitate the development of diagnostic and therapeutic strategies for the treatment of human cancer. Epigenetic changes associated with EMT are clinically relevant as a potential biomarker. Given the complexity of epigenetic transformations and, importantly, the need to balance the processes involved in epigenetic modifications within target DNA or histones, it remains open how to develop epigenetic treatment strategies aimed at restoring the balance of cancer cells/tissues with minimal impact on normal cells. So called *context-dependent behaviors* of epigenetic-regulated networks in cancer is crucial to understanding and proposing appropriate therapeutic solutions. Peripheral therapy does not seem to be an accurate solution, and the approach analogous to other therapeutic strategies, including the use of targeted therapy, has its justification. We believe that a detailed understanding of epigenetic aspects in EMT regulation and metastasis will undoubtedly be a good way to develop prognostic biomarkers in cancer, whereas the enzymatic basis of epigenetic changes could become a good research model for testing new epidrugs.

## Figures and Tables

**Table 1:** Examples of epigenetic regulatory mechanisms of epithelial and mesenchymal markers.

Gene	Mechanism of Action	Types of Cancer	Ref.
*CDH1*	DNA metylation	lung adenocarcinoma	[11]
	promoter DNA methylation	breast cancer	[12]
	enhanced H3K27me3, reduced H3K9ac, H3K4me3, H3K9me2	prostate cancer stem-like cells	[13]
	Overexpression of DNMT3Ab DNA hypermethylation, H3K9me2 and H3K27me3	gastric cancer	[14]
*CDH2*	DNA hypermethylation at CpG site near the AP-2 binding sequence	ductal carcinoma in situ	[15]
*FN1*	miR-9-3p	nasopharyngeal carcinoma	[16]
*MMP2*	DNA promoter methylation	breast cancer	[17]
	HDAC10 suppresses expression by interacting with the promoter regions and deacetylating histones H3 and H4	cervical cancer	[18]
*MMP9*	DNA promoter methylation H3K4me3	breast cancer	[19]
	miR-211	gastric cancer	[20]
	HDAC10 suppresses expression by interacting with the promoter regions and deacetylating histones H3 and H4	cervical cancer	[18]
*DSP*	DNA promoter methylation	lung cancer	[21]
	H3K9 demethylase	chondrosarcoma	[22]
*COL1*	DNA promoter methylation	hepatocellular carcinoma	[23]
*KRT13*	promoter abberant hypermethylation PRC2-mediated H3K27me3	oral squamous cel carcinoma (cell lines)	[24]
*KRT14*	H3K27me3 enhances transcription by attenuating binding of Sp1 to promoter	triple negative breast cancer	[25]
*KRT18*	DNA hypermethylation	brain metastasis of breast cancer	[26]
	histone H3 hypoacetylation	hepatocellular carcinoma	[27]
*KRT19*	promoter hypermethylation	hepatocellular carcinoma	[28]
*KRT8* *KRT19*	promoter hypomethylation	lung adenocarcinoma	[29]
*KRT23*	promoter hypomethylation	colon adenocarcinoma	[30]
*OCLN*	downregulation corelated with increase in miR-122 and miR-34a expression	clear cell renal carcinoma	[31]
*VIM*	promoter hypermethylation	cervical cancer cells	[32]
	DNA methylation (detected in the serum)	colorectal cancer at every disease stage	[33]
	low methylation associated with elevated vimentin protein level in the serum	breast cancer	[34]

## Data Availability

Not applicable.
